# Antihyperglycemic Effect of *Ginkgo biloba* Extract in Streptozotocin-Induced Diabetes in Rats

**DOI:** 10.1155/2013/162724

**Published:** 2012-12-20

**Authors:** Daye Cheng, Bin Liang, Yunhui Li

**Affiliations:** ^1^Department of Transfusion, The First Hospital of China Medical University, North Nanjing Street No. 155, Shenyang 110001, China; ^2^High Vocational Technological College, China Medical University, Shenyang 11001, China; ^3^Department of Clinical Laboratory, No. 202 Hospital, Shenyang 110003, China

## Abstract

The *Ginkgo biloba* extract (GBE) has been reported to have a wide range of health benefits in traditional Chinese medicine. The aim of this study was to evaluate the antihyperglycemic effects of GBE on streptozotocin- (STZ-) induced diabetes in rats. Diabetes was induced in male *Wistar* rats by the administration of STZ (60 mg/kg b.w.) intraperitoneally. GBE (100, 200, and 300 mg/kg b.w.) was administered orally once a day for a period of 30 days. Body weight and blood glucose levels were determined in different experimental days. Serum lipid profile and antioxidant enzymes in hepatic and pancreatic tissue were measured at the end of the experimental period. Significant decreases in body weight and antioxidant ability and increases in blood glucose, lipid profile, and lipid peroxidation were observed in STZ-induced diabetic rats. The administration of GBE and glibenclamide daily for 30 days in STZ-induced diabetic rats reversed the above parameters significantly. GBE possesses antihyperglycemic, antioxidant, and antihyperlipidemia activities in STZ-induced chronic diabetic rats, which promisingly support the use of GBE as a food supplement or an adjunct treatment for diabetics.

## 1. Introduction

Diabetes is a global epidemic with an estimated worldwide prevalence of 246 million people in 2007 and forecasts to rise to 300 million by 2025 [1]; consequently, diabetes presents a major challenge to healthcare systems around the world. Diabetes is a metabolic disorder of multiple etiologies characterized by chronic hyperglycemia with disturbance of carbohydrate, fat, and protein metabolism resulting from defects in insulin secretion, insulin action, or both [2]. Many oral antihyperglycemic agents, such as sulfonylurea and biguanides, are available along with insulin for the treatment of diabetes, but these agents have significant side effects, and some are ineffective in chronic diabetes patients [3]. Thus, there is an increasing need of new natural antihyperglycemic products especially nutraceuticals with less side effects, safe, and high antihyperglycemic potential.

Previous studies have demonstrated that diabetes exhibits enhanced oxidative stress and high reactive oxygen species (ROS) in pancreatic islets due to persistent and chronic hyperglycemia, thereby depletes the activity of antioxidative defense system, and thus promotes free radical generation [4]. A number of mechanisms or pathways by which hyperglycemia, the major contributing factor of increased ROS production, causes tissue damage or diabetic complications have been identified [5]. Also, reduced antioxidant levels as a result of increased free radical production in experimental diabetes have been reported [6]. The rise in free radical activity is suggested to play an important role in lipid peroxidation and protein oxidation of cellular structures resulting in cell injury and implicated in the pathogenesis of vascular disease which are the mainly cause of morbidity and mortality in diabetes [7]. Streptozotocin (STZ) is frequently used to induce diabetes in experimental animals through its toxic effects on pancreatic *β*-cells [8, 9] and as a potential inducer of oxidative stress. It has been reported that diabetes induced by STZ is the best characterized system of xenobiotic-induced diabetes and the commonly used model for the screening of antihyperglycemic activities.

Traditional medicines derived mainly from plants played an important role in the management of diabetes. *Ginkgo biloba* is a dioecious tree with a history of use in traditional Chinese medicine and has many pharmacologic effects. The mechanism of action of *Ginkgo* is believed to be linked with its functions as a neuroprotective agent, an antioxidant, a free-radical scavenger, a membrane stabilizer, and an inhibitor of the platelet-activating factor, and so on [10–13]. *Ginkgo biloba* extract (GBE) from *Ginkgo biloba* leaves is commonly used in dietary supplements for ailments and has showed excellent clinical effects in many cases. The goal of this study was to evaluate the effects of GBE on the antihyperglycemic ability in STZ-induced diabetes rats. Furthermore, the positive roles of natural products in the correction of oxidative stress and hyperlipidaemia, which are diabetes-related complication, were also assessed.

## 2. Materials and Methods

### 2.1. Materials

The powder form of GBE was purchased from Hangzhou Greensky Biological Tech (Hangzhou, China). All reagents used in this research were of analytical grade and obtained from Shenyang Biotechnology Co. Ltd.

### 2.2. Induction of Diabetes to Experimental Rats

Diabetes was induced by a single intraperitoneal injection of a freshly buffered (0.1 M citrate, pH 4.5) solution of STZ at a dosage of 60 mg/kg body weight (b.w.). After 72 h of STZ administration, the tail vein blood was collected to determine fasting blood glucose level. Only rats with fasting blood glucose over 250 mg/dL were considered diabetic and included in the experiments. Treatment with GBE started after the last STZ injection. Blood samples were drawn at 48 hours, 15 days, and 30 days till the end of the study (30 days).

### 2.3. Experimental Design

A total of 70 matured normoglycemic male *Wistar* rats (12–14 weeks of age, weighing about 180  ±  10 g) were collected for this experiment. Animals were acclimated for a period of 7 days in our laboratory condition prior to the experiment. The rats were fed with standard laboratory diet and allowed to drink water *ad libitum*. Animal experiments were carried out in accordance with institutional ethical guidelines for the care of laboratory animals of the China Medical University. 

Rats randomly selected were divided into seven groups, comprising ten rats each. The treatment schedule was as follows: (1) normal control group (N group), (2) STZ control group (D group), (3) GBE- (200 mg/kg b.w.) treated control group (N + G group), (4) GBE- (100 mg/kg b.w.) treated STZ group (D + LG group), (5) GBE- (200 mg/kg b.w.) treated STZ group (D + MG group), (6) GBE- (300 mg/kg b.w.) treated STZ group (D + HG group), and (7) glibenclamide- (5 mg/kg b.w.) treated STZ group (D + GLI group). The treatment with GBE and glibenclamide started after the last STZ injection, where the vehicle, GBE, and glibenclamide were administrated orally to the respective group rats. After 48 hours, 15 days, and 30 days of treatment, the rats were fasted overnight, and blood glucose and body weight were measured in the morning. The serum lipid profile was determined after 30 days of treatment. At the end of 30-day experiment, all rats were anesthetized with pentobarbital sodium (35 mg/kg) and euthanized by cervical decapitation. The liver and pancreas were excised immediately from the animals, washed with ice-chilled physiological saline, and stored at −80°C.

### 2.4. Determination of Serum Glucose and Lipid Profile

The serum concentrations of glucose, triglyceride (TG), total cholesterol (TC), high-density lipoprotein cholesterol (HDL-C), and low-density lipoprotein cholesterol (LDL-C) were determined using commercially available kits (BIOSINO Bio-technology and Science INC, China). 

### 2.5. Determination of Oxidative Stress Markers

The superoxide dismutase (SOD), glutathione peroxidase (GSH-Px), catalase (CAT) activities, and glutathione (GSH) level in hepatic and pancreatic tissue were measured using commercially available kits (Jiancheng Bioengineering Institute, Nanjing, China). Lipid peroxidation was measured as malondialdehyde (MDA) level in hepatic and pancreatic tissue according to Jain's method [14].

### 2.6. Statistical Analysis

Results are expressed as the means ± SD. Statistical analysis was performed by ANOVA for multiple comparisons (SPSS, Version 15.0). A value of *P* < 0.05 was considered significant.

## 3. Results

### 3.1. Effect of GBE on Body Weight in Experimental Groups

Body weights of rats in the seven groups were monitored during the experimental period. As shown in Table 1, there is no difference in the different groups before treatment and after 48 h (*P* > 0.05). Body weights of rats in D group were lower than those in other groups after 15 days and 30 days (*P* < 0.01). STZ caused a significant weight loss of rats in D group while treatment with GBE at different concentrations or glibenclamide suppressed the decrease in the body weight. 

### 3.2. Effect of GBE on Blood Glucose in Experimental Groups

**Figure 1:**
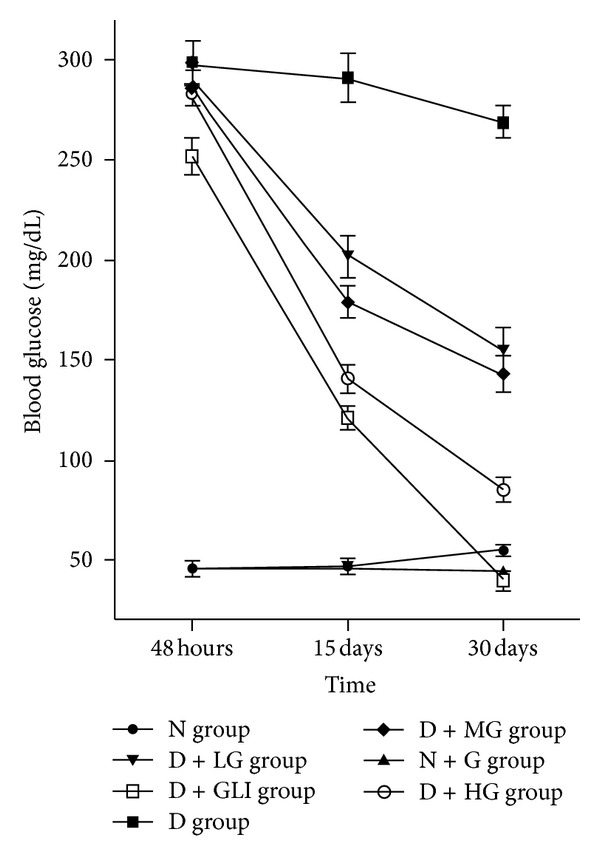
Changes of blood glucose of rats in the seven groups during the experimental period of 30 days. Values are mean ± SD (*n* = 10 animals).

The STZ-induced diabetic rats exhibited a significant increase in fasting blood glucose (299 ± 10 mg/dL) as compared to non-STZ-treated rats (49 ± 6 mg/dL) before the initial treatment of GBE or glibenclamide (*P* < 0.01). After GBE and glibenclamide treatment, the changes of blood glucose levels in different experimental groups were shown in Figure 1. At 48 hours, the levels of blood glucose in D group (298.3 ± 11.2 mg/dL), D + LG group (291.6 ± 8.8 mg/dL), D + MG group (286.4 ± 9.2 mg/dL), D + HG group (283.5 ± 5.6 mg/dL), and D + GLI group (252.9 ± 9.1 mg/dL) were higher than N group (46.8 ± 4.6 mg/dL) and N + G group (45.3 ± 3.3 mg/dL) (*P* < 0.01). The administration of GBE or glibenclamide for 30 days in D + LG group (155.5 ± 11.6 mg/dL), D + MG group (143.1 ± 9.6 mg/dL), D + HG group (85.0 ± 6.7 mg/dL), and D + GLI group (40.9 ± 5.4 mg/dL) caused a significant decrease in blood glucose levels when compared with D group (269.7 ± 8.4 mg/dL) (*P* < 0.05), but all GBE-treated group retained high blood glucose (>100 mg/dL) after 15 days. GBE caused a significant dose- (*P* < 0.01) and time-dependent reduction (*P* < 0.01) in blood glucose levels of diabetic rats. The blood glucose values of diabetic rates showed a tendency to normal levels after administration of GBE at 300 mg/kg b.w. and glibenclamide, 5 mg/kg b.w. in 30 days.

### 3.3. Effect of GBE on Serum Lipid Profiles in Experimental Groups

Our observation provides further support to the growing body of evidence showing that STZ-induced diabetes can also induce anomaly of serum TC, TG, HDL-C, and LDL-C.  Table 2 showed the levels of serum TG, TC, LDL-C, and HDL-C of rats in different experimental groups. Rats in D group displayed a significant increase in the levels of TG, TC, and LDL-C in comparison with N group (*P* < 0.01). However, serum HDL-C level of rats in D group was significantly lower than that of rats in N group (*P* < 0.01). Similar with the glibenclamide-treated STZ group, GBE administration showed a significant decrease in the levels of serum TG, TC, and LDL-C or a significant increase in the level of HDL-C after 30-day treatment when compared with D group.

### 3.4. Determination of Oxidative Stress Marker in Hepatic and Pancreatic Tissue

As shown in Table 3, a marked increase of MDA production and decrease of antioxidant level (GSH) and antioxidant enzyme activity (SOD, CAT, GSH-Px) were observed in the hepatic and pancreatic tissues of rats in D group when compared with N group (*P* < 0.01). GBE and glibenclamide treatment significantly inhibited the formation of MDA and raised antioxidant level (GSH) and antioxidant enzyme activity (SOD, CAT, GSH-Px) in a dose-dependent manner. Likewise, GBE exhibited the same antioxidation effects as glibenclamide at the dose of 5 mg/kg b.w.

## 4. Discussion

Diabetes is increasing at an alarming rate worldwide, which can mainly be attributed to the sedentary life style and calorie-rich diet. Diabetes is often linked with abnormal lipid metabolism and is considered as a major factor for the development of atherosclerosis and cardiovascular complication [15]. Recently, the WHO Expert Committee recommended the importance to investigate and explore hypoglycemic agents from plant origin because plants used in the traditional medicine have fewer side effects than synthetic drugs [16]. Currently, GBE is widely used for medicine in China and showed excellent clinical effects in many aspects, and the pharmacological mechanisms including modification of Ca^2+^ signaling [17], clearing oxygen free radical [18], decreasing lipid peroxidation, and promoting the synthesis and release of epoprostenol [6]. Increasing evidence has demonstrated that GBE has a potential efficacy in glucose metabolism and lipid metabolism. Therefore, the present study was aimed to assess the effect of GBE on hyperglycemia, lipid profile, and enzymatic and nonenzymatic antioxidants in STZ-induced diabetic rats.

In our study, Figure 1 shows the changes in body weight of normal and streptozotocin-induced diabetic rats. STZ-injection-induced diabetes is associated with the characteristic loss of body weight which is due to increased muscle wasting and due to loss of tissue proteins [19]. As expected in D group, the body weight of rats was progressively reduced, and the treatment of diabetic rats with glibenclamide and GBE improved body weight significantly, which indicates the prevention of muscle tissue damage due to hyperglycemic condition. In addition, STZ injection caused diabetes, which may be due to destruction of *β*-cells of the islets of Langerhans [20]. G. B. Kudolo has already reported that ingestion of 120 mg of GBE as a single dose for 3 months for individuals leads to an increase in pancreatic *β*-cell function [21, 22]. Zhou et al. proposed that GBE improved insulin sensitivity mainly by enhancing insulin receptor substrate 2 transcription and preventing insulin resistance. Similarly, our data showed that the daily administration of GBE for 30 consecutive days abolished the blood glucose increase in the STZ-induced diabetic rats. This effect was dose dependent and time dependent. The reduction of blood glucose may be either due to the increased level of plasma insulin in diabetic rats, which may influence the stimulation of pancreatic insulin secretion from *β*-cells in islets of Langerhans, or due to the enhanced transport of blood glucose to peripheral tissue. Our results supported the reported evidence that GBE has the potential to prevent insulin resistance and is a promising antidiabetic drug [23].

Previous studies have demonstrated that GBE exerts multidirectional lipid-lowering effects on the rat metabonome, including limitation of the absorption of cholesterol, inactivation of HMG-CoA, and favorable regulation of profiles of essential polyunsaturated fatty acid [24]. In diabetes, hyperglycemia is accompanied with dyslipidemia [25] characterized by increase in TC, LDL, VLDL, and TG and fall in HDL. This altered serum lipid profile was reversed towards normal after treatment with GBE. The possible mechanism through which GBE exerts its anti-hyperlipidemic effect might include the changed activity of cholesterol biosynthesis enzymes and/or the changed level of lipolysis which are under the control of insulin [26]. It is reported that GBE treatment could decrease the capacity of LDL to carry free cholesterol to various tissues without affecting the capacity of HDL to carry cholesterol back to the liver in rats [27]. In addition, GBE treatment can partially reverse ethanol-induced dyslipidemia at dose levels of 48 and 96 mg/kg b.w. in rats by reducing the lipid peroxidation induced by ethanol [28]. Our results indicated that the lipid-lowering effect of GBE could be an indirect consequence of amelioration of insulin resistance or direct hypolipidemic effect mediated through other mechanisms [29].

Hyperglycemia is a main cause for elevated free radical levels, followed by production of ROS, which can lead to increased lipid peroxidation and altered antioxidant defense and further impair glucose metabolism in biological system [30]. An imbalance between oxidation and antioxidant status has been shown to play an important role in mediating insulin resistance [29]. Overwhelming free radicals generated due to oxidative stress may develop several adverse effects commonly seen in diabetes such as neuropathy, nephropathy, retinopathy, and vascular disorders [31]. The major antioxidant enzymes, including SOD, CAT, and GSH-Px, are regarded as the first line of the antioxidant defense system against ROS generated in vivo during oxidative stress and act cooperatively at different sites in the metabolic pathway of free radicals [32]. In our study, reduced activities of SOD, CAT, and GSH-Px in the liver and pancreas have been observed in diabetic rats. The administration of GBE for 30 days increased the SOD, CAT, and GSH-Px activity and GSH level in the liver and pancreas of diabetic rats. Robertson et al. demonstrated that antioxidants have been shown to brake the worsening of diabetes by improving *β*-cells function in animal models and suggested that enhancing antioxidant defense mechanisms in pancreatic islets may be a valuable pharmacologic approach to managing diabetes [33]. Modak MA et al. reported that the control of hyperglycemia leads to improvement in oxidative stress profile, and enhancing antioxidant defense mechanisms in pancreatic islets helps them to cope better with oxidative stress. Since GBE is a complex mixture of ingredients with a unique broad spectrum of pharmacological activities, it probably acts through several different mechanisms covering ROS scavenger and/or enhancing antioxidant ability. Moreover, Naik et al. have demonstrated that GBE is scavengers of free radicals by increasing levels of free radical scavenging enzymes [34]. It is also reported that GBE may reduce the oxidative stress in the reperfused myocardium and increase the antioxidant activity in ischemia reperfusion rats. Further, lipid peroxidation measurement is a more practical and safer method to evaluate the factors causing cellular injury and the activation of the common pathway. Tissue MDA content, the final product of lipid breakdown caused by oxidative stress, is an important indicator of free radical-induced lipid peroxidation [35–37]. GBE-treated rats showed decreased level of MDA, suggesting that GBE has antioxidant capacity. 

## 5. Conclusion

In summary, GBE possesses antihyperglycemic, antioxidant, and antihyperlipidemic activities in STZ-induced chronic diabetic rats, which promisingly support the use of GBE as a food supplement or an adjunct treatment for diabetics. Moreover, further work is necessary to elucidate in detail the mechanism of action of the GBE at the cellular and molecular levels.

## Figures and Tables

**Table 1:** Changes of the body weight (g) of rats in the seven groups during the experimental period of 30 days.

	Before treatment	48 h	15 days	30 days
N group	178.3 ± 5.6	183.4 ± 6.5	214.3 ± 7.7	220.3 ± 6.3
D group	180.1 ± 6.9	177.3 ± 5.9	174.1 ± 10.2^a^	169.3 ± 11.6^a^
N + G group	176.9 ± 5.8	186.1 ± 8.8	209.6 ± 9.6^b^	220.8 ± 10.0^b^
N + LG group	179.3 ± 7.6	183.0 ± 7.2	187.6 ± 5.5^a,b^	206.3 ± 5.9^a,b^
N + MG group	177.4 ± 5.1	180.6 ± 4.6	190.6 ± 4.9^a,b^	213.6 ± 9.9^b^
N + HG group	178.6 ± 7.7	186.9 ± 5.8	199.5 ± 7.1^a,b^	216.2 ± 7.8^b^
N + GLI group	180.2 ± 6.3	187.9 ± 5.0	196.3 ± 6.1^a,b^	212.1 ± 11.8^b^

Values are means ± SD for 10 rats in each group. N group: normal control; D group: diabetes group; N + G group: normal control plus GBE; D + LG group: diabetes plus low GBE treatment; D + MG group: diabetes plus middle GBE treatment; D + HG group: diabetes plus high GBE treatment; D + GLI group: diabetes plus glibenclamide treatment.

^a^Indicates statistical significance of *P* < 0.01 compared to N group; ^b^ *P* < 0.01 compared to D group.

**Table 2:** Effect of GBE treatment on serum lipid profile of rats in experimental groups.

	N group	D group	N + G group	D + LG group	D + MG group	D + HG group	D + GLI group
TG (mmol/L)	1.48 ± 0.35	2.32 ± 0.51^a^	1.46 ± 0.38^b^	1.75 ± 0.40^c^	1.65 ± 0.41^b^	1.52 ± 0.28^b^	1.36 ± 0.40^b^
TC (mmol/L)	1.69 ± 0.26	2.71 ± 0.48^a^	1.61 ± 0.30^b^	2.02 ± 0.51^b^	1.83 ± 0.44^b^	1.77 ± 0.33^b^	1.65 ± 0.31^b^
HDL-C (mmol/L)	1.35 ± 0.23	0.92 ± 0.30^a^	1.21 ± 0.28^b^	0.98 ± 0.25	1.19 ± 0.23^c^	1.28 ± 0.30^c^	1.20 ± 0.22^b^
LDL-C (mmol/L)	0.56 ± 0.09	0.73 ± 0.11^a^	0.51 ± 0.08^b^	0.68 ± 0.09	0.67 ± 0.03	0.62 ± 0.06^c^	0.57 ± 0.10^b^

Values are means ± SD for 10 rats in each group. N group: normal control; D group: diabetes group; N + G group: normal control plus GBE; D + LG group: diabetes plus low GBE treatment; D + MG group: diabetes plus middle GBE treatment; D + HG group: diabetes plus high GBE treatment; D + GLI group: diabetes plus glibenclamide treatment.

^a^Indicates statistical significance of *P* < 0.01 compared to N group; ^b^ *P* < 0.01 compared to D group.

^c^ *P* < 0.05 compared to D group.

**Table 3:** Effect of GBE treatment on hepatic and pancreatic oxidative stress markers of rats in experimental groups.

	N group	D group	N + G group	D + LG group	D + MG group	D + HG group	D + GLI group
Liver							
GSH (*μ*g/mg protein)	512.36 ± 91.25	365.50 ± 84.77^a^	529.36 ± 101.23^b^	400.28 ± 79.46	451.33 ± 89.44^c^	486.36 ± 98.31^b^	482.09 ± 106.30^c^
CAT (U/mg protein)	362.63 ± 18.52	302.05 ± 22.82^a^	374.47 ± 26.36^b^	313.25 ± 24.47	322.52 ± 17.52^c^	349.96 ± 25.01^b^	344.80 ± 20.91^b^
SOD (U/mg protein)	542.35 ± 24.21	235.27 ± 34.22^a^	602.0 ± 40.50^b^	322.21 ± 32.65^b^	414.11 ± 35.33^b^	505.20 ± 41.52^b^	523.36 ± 36.66^b^
GSH-Px (U/mg protein)	3023 ± 217	2236 ± 259^a^	3111 ± 285^b^	2567 ± 198^b^	2758 ± 252^b^	2904 ± 220^b^	2808 ± 244^b^
MDA (nmol/mg protein)	9.03 ± 1.25	16.91 ± 1.81^a^	8.85 ± 1.33^b^	14.28 ± 1.57^b^	10.05 ± 1.01^b^	9.66 ± 1.41^b^	10.36 ± 1.55^b^
Pancreas							
GSH (*μ*g/mg protein)	444.08 ± 71.44	294.14 ± 69.27^a^	408.50 ± 66.85^b^	312.58 ± 75.55	360.00 ± 57.19^c^	405.81 ± 63.36^b^	406.16 ± 56.39^b^
CAT (U/mg protein)	298.02 ± 21.30	174.22 ± 14.52^a^	287.34 ± 22.71^b^	196.35 ± 18.90^b^	209.42 ± 23.00^b^	212.56 ± 20.66^b^	274.30 ± 21.94^b^
SOD (U/mg protein)	415.00 ± 33.20	197.38 ± 36.55^a^	385.52 ± 32.11^b^	222.44 ± 36.40	356.78 ± 37.80^b^	379.91 ± 38.92^b^	364.82 ± 40.24^b^
GSH-Px (U/mg protein)	658 ± 89	462 ± 92^a^	711 ± 101^b^	555 ± 74^c^	596 ± 94^b^	623 ± 78^b^	539 ± 79^b^
MDA (nmol/mg protein)	8.77 ± 1.03	14.91 ± 1.44^a^	8.05 ± 1.11^b^	12.50 ± 1.48^b^	10.87 ± 1.61^b^	9.22 ± 1.31^b^	8.99 ± 1.40^b^

Values are means ± SD for 10 rats in each group. N group: normal control; D group: diabetes group; N + G group: normal control plus GBE; D + LG group: diabetes plus low GBE treatment; D + MG group: diabetes plus middle GBE treatment; D + HG group: diabetes plus high GBE treatment; D + GLI group: diabetes plus glibenclamide treatment.

^a^Indicates statistical significance of *P* < 0.01 compared to N group; ^b^ *P* < 0.01 compared to D group.

^c^ *P* < 0.05 compared to D group.
